# Enhanced pinocembrin production in *Escherichia coli* by regulating cinnamic acid metabolism

**DOI:** 10.1038/srep32640

**Published:** 2016-09-02

**Authors:** Weijia Cao, Weichao Ma, Xin Wang, Bowen Zhang, Xun Cao, Kequan Chen, Yan Li, Pingkai Ouyang

**Affiliations:** 1 State Key Laboratory of Materials-Oriented Chemical Engineering, Nanjing 211816, P.R. China; 2 College of Biotechnology and Pharmaceutical Engineering, Nanjing Tech University, Nanjing 211816, P.R. China; 3 College of Bioengineering and Biotechnology, Tianshui Normal University, Tianshui 741001, P.R. China

## Abstract

Microbial biosynthesis of pinocembrin is of great interest in the area of drug research and human healthcare. Here we found that the accumulation of the pathway intermediate cinnamic acid adversely affected pinocembrin production. Hence, a stepwise metabolic engineering strategy was carried out aimed at eliminating this pathway bottleneck and increasing pinocembrin production. The screening of gene source and the optimization of gene expression was first employed to regulate the synthetic pathway of cinnamic acid, which showed a 3.53-fold increase in pinocembrin production (7.76 mg/L) occurred with the alleviation of cinnamic acid accumulation in the engineered *E. coli*. Then, the downstream pathway that consuming cinnamic acid was optimized by the site-directed mutagenesis of chalcone synthase and cofactor engineering. S165M mutant of chalcone synthase could efficiently improve the pinocembrin production, and allowed the product titer of pinocembrin increased to 40.05 mg/L coupled with the malonyl-CoA engineering. With a two-phase pH fermentation strategy, the cultivation of the optimized strain resulted in a final pinocembrin titer of 67.81 mg/L. The results and engineering strategies demonstrated here would hold promise for the titer improvement of other flavonoids.

Flavonoids are plant secondary metabolites, which have been of special interest due to their health-promoting benefits and their applications in the food and pharmaceutical industries^1^. The pharmaceutical functions of these compounds have been linked to lower risks of many chronic diseases including cancer, cardiovascular disease, chronic inflammation and many other degenerative diseases^2^,^3^. Pinocembrin (5,7-dihydroxyflavanone) shows a variety of biological properties and is one of the primary flavonoids isolated from a variety of plants^4^,^5^,^6^. Currently, pinocembrin is primarily obtained by extraction from plants, which is tedious and inefficient. Chemical syntheses also suffer from the demand for expensive substrates, catalyst instability and the production of isomers^7^,^8^. On the contrary, biosynthesis has several advantages, including the utilization of environmentally friendly feedstock, low energy requirements and low waste emissions. Therefore, biosynthesis-based pinocembrin production has emerged as an attractive approach^9^.

Pinocembrin can be biosynthesized from the aromatic amino acid L-phenylalanine through four enzymatic steps (Fig. 1)^10^. L-phenylalanine is first deaminated to yield cinnamic acid by the action of phenylalanine ammonia lyase (PAL). The resulting cinnamic acid is converted to pinocembrin chalcone by 4-coumarate-CoA ligase (4CL) and chalcone synthase (CHS). Finally, pinocembrin chalcone is rapidly converted to pinocembrin under alkaline conditions or by chalcone isomerase (CHI) (Fig. 1). A number of studies have been published on the production of pinocembrin in *Escherichia coli*^7^,^11^,^12^. However, because a multiple-gene pathway is required for the biosynthesis, the final yield of the compound is often limited by several deleterious effects, such as the low levels of available precursors or cofactors, the metabolic burden of heterologous protein production, or the accumulation of pathway intermediates that are inhibitory to the host^13^,^14^,^15^. To achieve a high yield of pinocembrin, researchers have focused mainly on redirecting the carbon flux to pathways responsible for the generation of malonyl-CoA in *E. coli*^9^,^12^,^16^,^17^. In our previous study, we also found that overexpression of β-ketoacyl-ACP synthase II (FabF) could increase cellular malonyl-CoA levels and pinocembrin production^18^. However, this was accompanied by an accumulation of the intermediate, cinnamic acid^18^. In this work, which used L-phenylalanine as an additive, we discovered that growth inhibition and a low pinocembrin titer were correlated with the accumulation of cinnamic acid, which has not been found before.

Previous studies have demonstrated that imbalances within metabolic pathways could lead to the accumulation of intermediate metabolites in multiple-gene pathways^19^. To address this problem, the combination of metabolic engineering and protein engineering has offered tremendous opportunities for customized optimization of multi-gene pathways^7^,^20^. For example, Zhao and coworkers screened 18 gene combinations to decrease the dihydroquercetin accumulation and obtained a titer of 130.8 mg/L of catechin from 300 mg/L eriodictyol^21^. During naringenin biosynthesis, two additional copies of the CHS gene were introduced to alleviate the bottleneck downstream of coumaric acid, resulting in a 2.5-fold increase in naringenin production^14^. Similarly, changes in various promoters and gene copy numbers were combined to balance the upstream and downstream modules to produce a maximum titer of 1.02 g/L taxadiene^22^. Through the *in vivo* evolution of stilbene synthase, the pinosylvin production was increased, which was accompanied by decreased accumulation of cinnamic acid^8^,^23^.

In the present report, we found that the accumulation of cinnamic acid was disadvantageous for the pinocembrin production in *E. coli*. By screening heterologous gene sources of PAL and 4CL, we reduced the cinnamic acid level and obtained the optimized combination. Furthermore, PAL and 4CL were arranged into different plasmids to decrease the biosynthesis of cinnamic acid. Meanwhile, Site-directed mutagenesis of CHS and malonyl-CoA engineering strategy enabled further consumption of cinnamic acid and increase of pinocembrin production in *E. coli.*

## Results

### The effect of cinnamic acid accumulation on pinocembrin synthesis

**Figure 2:**
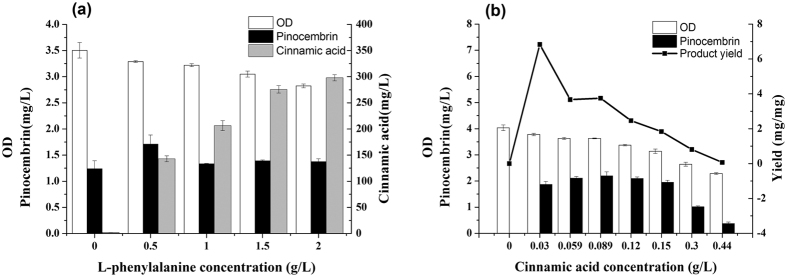
The effect of L-phenylalanine or cinnamic acid supplement on biomass and product distribution. **(a)** The engineered strain carrying pET-YEPAL-SE4CL and pRSF-CHS-CHI cultured with different L-phenylalanine concentration ranging from 0 g/L to 2 g/L. Black bars: pinocembrin (mg/L); gray bars: cinnamic acid (mg/L); white bars: OD_600_. **(b)** The engineered strain carrying pET-SE4CL and pRSF-CHS-CHI cultured with different cinnamic acid concentration ranging from 0 g/L to 0.44 g/L. Black bars: pinocembrin (mg/L); gray bars: cinnamic acid (mg/L); black line: the pinocembrin yield (mg/mg). The error bars indicate the standard deviation, as determined from triplicate experiments (three independent bacterial cultures).

To induce the production of pinocembrin in *E. coli*, PAL from *Rhodotorula mucilaginosa*^24^,^25^, 4CL from *Streptomyces coelicolor*A3 (2)^8^,^26^, CHS from *Glycyrrhiza uralensis*^27^ and CHI from *Medicago sativa*^9^,^12^ were both expressed in strain BL21 (DE3). As shown in Fig. 2a, the BL21 (DE3) strain was successfully transformed with pET-YEPAL-SE4CL and pRSF-CHS-CHI. Furthermore, the different concentrations of L-phenylalanine ranging from 0 g/L to 2.00 g/L were added to identify the most suitable substrate concentration for pinocembrin production. When the cultures were supplemented with 0.50 g/L phenylalanine, the strain exhibited the maximum pinocembrin production of 1.71 mg/L. When the phenylalanine concentration was increased to 1.00 g/L, the pinocembrin production was unexpectedly decreased, but the level of cinnamic acid was significantly increased (*p* < 0.05). With a further increase in phenylalanine concentration, the cinnamic acid level accumulated in the engineered strain was gradually increased and the pinocembrin production remained nearly unchanged. The OD_600_ of the engineered strain was also decreased by the accumulation of cinnamic acid, and the final OD_600_ was 2.82 in the cultures supplemented with 2.00 g/L phenylalanine.

Based on the above results, we speculated that the excessive accumulation of cinnamic acid *in vivo* might have a negative effect on pinocembrin production. To investigate the effects of cinnamic acid on pinocembrin production, the strain carrying pET-SE4CL and pRSF-CHS-CHI was constructed. As shown in Fig. 2b, different concentrations of cinnamic acid ranging from 0 g/L to 0.44 g/L were added to the cultures to identify the suitable substrate concentration that permitted pinocembrin production. In the presence of 0.03 g/L cinnamic acid, the strain exhibited a maximum pinocembrin yield (6.83%). On the other hand, the strain displayed the highest pinocembrin production of 2.19 mg/L in the presence of 0.089 g/L cinnamic acid. Any further increase in the cinnamic acid concentration led to a decrease in the pinocembrin production. When the cinnamic acid concentration was increased to 0.44 g/L, the pinocembrin production was only 0.37 mg/L with a yield of 0.06%. Meanwhile, the OD_600_ of the engineered strain was decreased as the cinnamic acid concentration increased, and the final OD_600_ was 2.29 in the presence of 0.44 g/L cinnamic acid.

### Optimization of the heterologous gene sources and expression to alleviate cinnamic acid accumulation

To alleviate the cinnamic acid overaccumulation, PALs and 4CLs from microorganisms and plants were screened for their effects. In this study, red yeast *Rhodotorula mucilaginosa* (*YE*PAL) and plant *Bambusa oldhamii* (*BO*PAL) were selected as the source of PAL expression because they have been reported to have different enzyme activity for L-phenylalanine^28^,^29^. Furthermore, we expressed the 4CL from *Streptomyces coelicolor* (*SE*4CL) and *Petroselinum crispum* (*PA*4CL) because SE4CL favors cinnamic acid over *p*-coumaric acid as a substrate, and *PA*4CL has a more diverse substrate specificity than other 4CL enzymes^26^,^30^. CHS and CHI were cloned into the pRSFDuet-1 vector under the individual control of a T7 promoter, while different gene sources of PAL and 4CL were cloned into the pETDuet-1 vector. Therefore, four separate plasmids (pET-*YE*PAL-*SE*4CL, pET-*YE*PAL-*PA*4CL, pET-*BO*PAL-*SE*4CL and pET-*BO*PAL-*PA*4CL) were constructed as described in supplementary material and introduced into competent BL21 (DE3) cells harbored pRSF-CHS-CHI. Although the engineered strain carrying pET-*YE*PAL-*SE*4CL and pET-*YE*PAL-*PA*4CL accumulated 142.95 mg/L and 63.62 mg/L cinnamic acid, the strain carrying pET-*BO*PAL-*SE*4CL and pET-*BO*PAL-*PA*4CL showed reduced cinnamic acid level of 20.30 mg/L and 26.07 mg/L, respectively. In addition, the combination of the PAL from *Bambusa oldhamii* and 4CL from *Petroselinum crispum* proved to be the most productive pathway for pinocembrin biosynthesis, but only 2.34 mg/L pinocembrin could be detected (Table 1).

**Figure 3:**
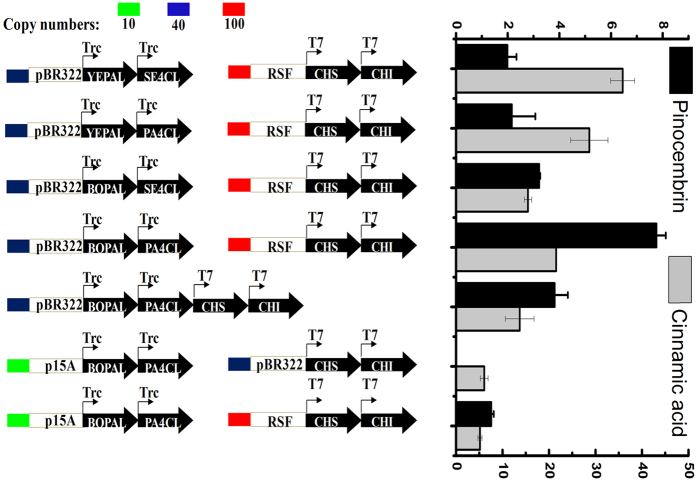
Optimization of pinocembrin and cinnamic acid production through varying plasmid promoter and copy number. RSF: origin of pRSFDuet-1; pBR322: origin of pTrc99a; p15A: origin of pACYCDuet-1; T7: T7 promoter; Trc: Trc promoter. Green rectangle: the low-copy-number of p15A (10); blue rectangle: the medium-copy-number of pBR322 (40); red rectangle: the high-copy-number of RSF (100); black bars: pinocembrin (mg/L); gray bars: cinnamic acid (mg/L). The error bars indicate the standard deviation, as determined from triplicate experiments (three independent bacterial cultures).

To further decrease the cinnamic acid level, the expression levels of PAL and 4CL were altered by introducing the pTrc99a, in which the T7 promoter of pETDuet-1was replaced by a trc promoter and the copy number of the plasmids was the same (50 copies per cell). As we expected, the strains carrying pTrc-*YE*PAL-*SE*4CL, pTrc-*YE*PAL-*PA*4CL, pTrc-*BO*PAL-*SE*4CL and pTrc-*BO*PAL-*PA*4CL produced 35.84 mg/L, 28.68 mg/L, 15.47 mg/L and 21.53 mg/L cinnamic acid, respectively. The engineered strains produced 1.99 mg/L, 2.16 mg/L and 3.22 mg/L pinocembrin by using pTrc-*YE*PAL-*SE*4CL, pTrc-*YE*PAL-*BO*PAL and pTrc-*BO*PAL-*SE*4CL, respectively. Notably, the strain carrying pTrc-*BO*PAL-*PA*4CL led to a three-fold increase in the pinocembrin titer (up to 7.76 mg/L) (Fig. 3). To determine whether the further decrease of cinnamic acid level had a positive effect on pinocembrin production, PAL and 4CL were cloned into the low-copy-number plasmids. When pACYC-Trc-*BO*PAL-Trc-*PA*4CL and pRSF-CHS-CHI were biotransformed into the *E. coli*, the engineered strain only produced 1.36 mg/L pinocembrin and 5.16 mg/L cinnamic acid. The strain carrying pTrc-4GS produced 3.82 mg/L pinocembrin and 13.69 mg/L cinnamic acid. Moreover, no pinocembrin could be detected when the strain harbored pACYC-Trc-*BO*PAL-Trc-*PA*4CL and pET-CHS-CHI. These results further suggested the accumulation of cinnamic acid would be adverse for pinocembrin production. Meanwhile, an appropriate cinnamic acid level in *E. coli* might be important for pinocembrin biosynthesis. Here, the strain carrying pTrc-*BO*PAL-*PA*4CL exhibited the higher production and was used for the further investigation in the following study.

### Rational design of CHS to improve the CHS activity

**Figure 4:**
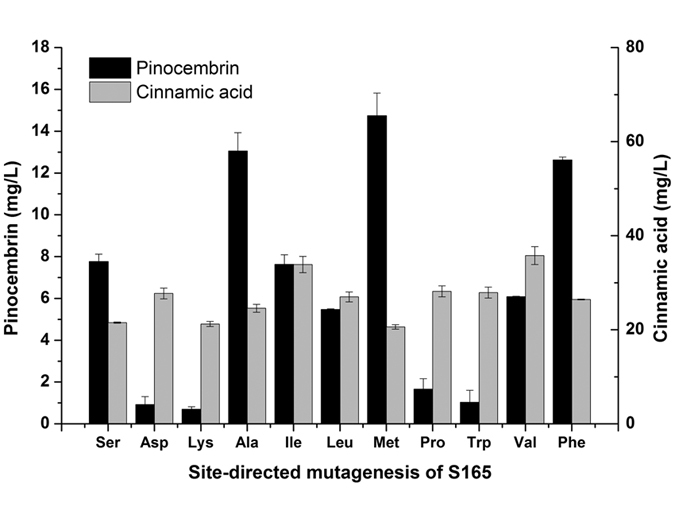
Optimization of pinocembrin and cinnamic acid production through site-directed mutagenesis of CHS. Ser: the control strain; Asp: the mutant strain S165D; Lys: the mutant strain S165K; Ala: the mutant strain S165A; Ile: the mutant strain S165I; Leu: the mutant strain S165L; Met: the mutant strain S165M; Pro: the mutant strain S165P; Trp: the mutant strain S165W; Val: the mutant strain S165V; Phe: the mutant strain S165F; Black bars: pinocembrin (mg/L); gray bars: cinnamic acid (mg/L). The error bars indicate the standard deviation, as determined from triplicate experiments (three independent bacterial cultures).

We hypothesized that poor enzyme activity of CHS might limit the consumption of cinnamic acid, further affecting the biosynthesis of pinocembrin. With the aim of improving CHS activity for enhanced pinocembrin biosynthesis, we consulted the web-based service PROSITE to analyze the functional sites of known CHS enzymes. As a result, a conserved motif (RLMMYQQGCFAGGTVLR) of CHS was identified. The F165 of motif was observed in most plants, including *Petunia hybrid*, *Medicago sativa* and *Arabidopsis thaliana* (Fig. 4). However, the nonpolar amino acid F165 was replaced by the polar amino acid S165 in *Glycyrrhiza uralensis*. Therefore, site-directed mutagenesis of CHS was conducted *in vivo.* As shown in Fig. 4, Ser was the control strain. The mutant strains S165D and S165K produced pinocembrin less than 1 mg/L. However, the mutant strains S165A, S165M and S165F showed positive effect on pinocembrin production, which represented a 68%, 90% and 63% increase compared with Ser, respectively. Meanwhile, the mutant trains S165I resulted in almost the same pinocembrin production. The mutant strains S165L, S165P, S165W and S165V led to a negative effect on pinocembrin production compared to Ser. Interestingly, the mutant strains S165A and S165F produced 24.59 and 26.44 mg/L cinnamic acid, which was an increase of 14% and 23% compared with Ser, respectively. The mutant strain S165M produced 20.62 mg/L cinnamic acid, which was nearly the same as that produced by Ser.

### Engineering malonyl-CoA availability to alleviate cinnamic acid accumulation

Malonyl-CoA availability is the rate-limiting step of CHS activity, which affects the metabolism of cinnamic acid. Three strategies were used in an effort to increase the availability of malonyl-CoA for pinocembrin biosynthesis in the mutant strain S165M. These strategies included the overexpression of acetyl-CoA carboxylase (ACC) from *Corynebacterium glutamicum*, the co-overexpression of acetyl-CoA synthase (encoded by *acs*) from *E. coli* with ACC, and the overexpression of β-ketoacyl-ACP synthase II (FabF) from *E. coli*. To understand the importance of the intracellular malonyl-CoA pool, the empty vector was introduced into BL21 (DE3) cells, and the relative levels of malonyl-CoA were compared for the different strategies. Overexpression of ACC alone increased the cellular malonyl-CoA level by 117% over that in wild-type *E. coli*. Co-overexpression of ACC and *acs* increased the intracellular malonyl-CoA level by 151% over that in the WT strain (Table 2). When FabF was overexpressed in engineered strain, the intracellular malonyl-CoA level increased by 68% over that in WT *E. coli*. A combination of the three manipulations gave a total increase in the intracellular malonyl-CoA level of 232% over that in WT *E. coli* (Table 2).

**Figure 5:**
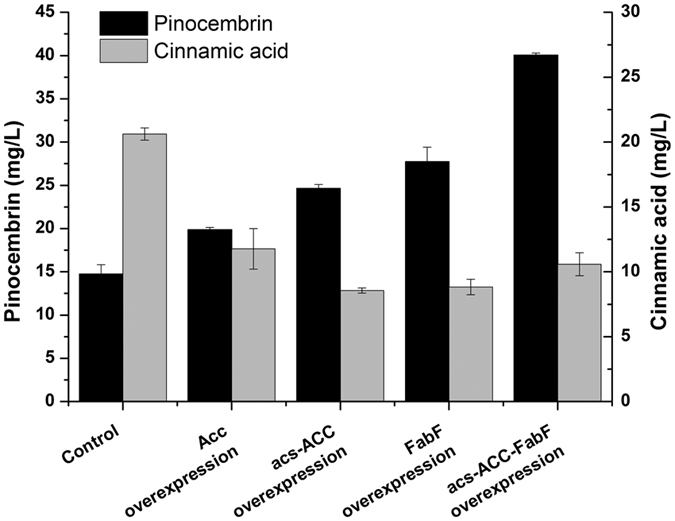
The effects of malonyl-CoA engineering strategies on the cinnamic acid and pinocembrin production. Control: the mutant strain S165M; Acc overexpression: the mutant strain S165M carrying pRSF-ACC, acs-ACC overexpression: the mutant strain S165M carrying pRSF-acs-ACC; FabF overexpression: the mutant strain S165M carrying pACYC-FabF; acs-ACC-FabF overexpression: the mutant strain S165M carrying pRSF-acs-ACC and pACYC-FabF. Black bars: pinocembrin (mg/L); gray bars: cinnamic acid (mg/L). The error bars indicate the standard deviation, as determined from triplicate experiments (three independent bacterial cultures).

Furthermore, as shown in Fig. 5, the overexpression of ACC alone increased the pinocembrin production by 35% over that in the mutant strain S165M. Co-overexpression of *acs* with ACC increased pinocembrin production to 24.63 mg/L. Overexpression of FabF alone enhanced the pinocembrin production by 88% over that in the mutant strain S165M. A combination of the three manipulations could produce 40.05 mg/L pinocemnbrin.

In addition, overexpression of ACC alone decreased the cinnamic acid production by 75% compared to that in the mutant of S165M strain. Co-overexpression of *acs* with ACC decreased the cinnamic acid production to 8.55 mg/L, which was a 141% decrease compared to that in the mutant strain S165M. The overexpression of FabF alone decreased the cinnamic acid production by 134% compared to that in the mutant strain S165M. A combination of the three manipulations led to a cinnamic acid level of 10.59 mg/L.

### Effects of two-phase pH control of batch fermentation on the production of cinnamic acid and pinocembrin

**Figure 6:**
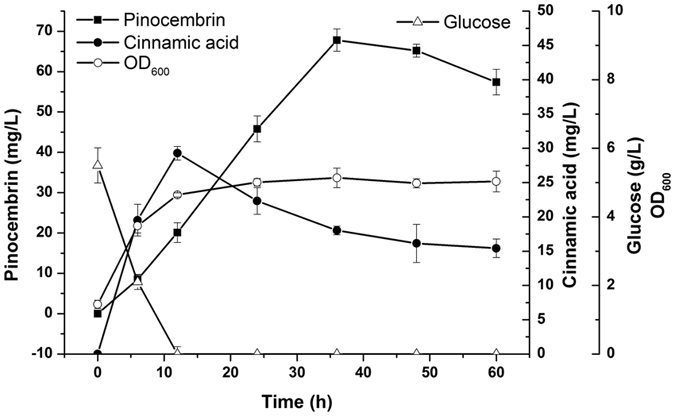
Time course of the changes in the OD600, glucose level and the production of pinocembrin and cinnamic acid during two-phase batch fermentation. Symbols used in the figure are as follows: OD_600_ (empty circles), glucose (empty triangles), cinnamic acid (filled circles) and pinocembrin (filled squares). The error bars indicate the standard deviation, as determined from triplicate experiments (three independent bacterial cultures).

To further characterize the optimized strain (S165M, *acs*↑, ACC↑, FabF↑), the strain was cultured in a 1.4-L batch bioreactor with two-phase pH control. The pH of the first phase was maintained at 7.0 from 0–12 h, and then the pH of the second phase was controlled at 6.5 from 12–48 h. During the first phase, glucose was totally exhausted and the biomass was increased. The level of cinnamic acid reached a peak of 29.28 mg/L at 12 h. This phase was beneficial for the expression of the upstream enzyme. As time goes by, the cinnamic acid production decreased and was 15.42 mg/L at 48 h, indicating that most cinnamic acid was converted to pinocembrin during this second phase. This pH was more suitable for the catalysis of the downstream enzyme. As shown in Fig. 6, the maximum pinocembrin production (67.81 mg/L) was obtained at 36 h. From 12 h to 36 h, a total of 47.70 mg/L pinocembrin was obtained.

## Discussion

As a basic scaffold of flavonoids, the phenylpropanoid pinocembrin showed promising biological activities^7^. Synthetic biology has become a beneficial tool to generate plant flavonoid products in a heterologous host^31^,^32^. Because low levels of intracellular malonyl-CoA could be a bottleneck that prevents efficient flavonoid production, there have been many investigations focused on malonyl-CoA engineering^15^,^16^,^33^,^34^. However, pinocembrin production was shown to be accompanied by cinnamic acid overaccumulation^18^. In the biosynthetic pathway of pinocembrin, cinnamic acid is a connecting note. This study demonstrated that the pinocembrin titer was negatively correlated with the overaccumulation of cinnamic acid. We also systematically demonstrated that this disadvantageous cinnamic acid overaccumulation can be alleviated based on step-wise metabolic engineering efforts, leading to improved pinocembrin production. The strategies included the screening of gene sources, the optimization of modular constructs, protein engineering and improvement of the malonyl-CoA level.

Most flavonoid-producing strains require the supplementation of phenylpropanoid intermediates^8^. In our study, L-phenylalanine was also added to the whole pinocembrin biosynthesis pathway as a precursor. To verify the effects of cinnamic acid overaccumulation on *E. coli* fermentation, experiments were performed using different amounts of L-phenylalanine or cinnamic acid added to the culture. Fermentation studies demonstrated that there is an obvious correlation between the cinnamic acid and pinocembrin production within a certain range (Fig. 2). The pinocembrin production and biomass were decreased as cinnamic acid accumulated, in spite of the addition of L-phenylalanine or cinnamic acid. The accumulation of cinnamic acid could be alleviated by reducing its biosynthesis and increasing its consumption.

First, we investigated the effect of four gene (PAL and 4CL) combinations on the cinnamic acid and pinocembrin production. PALs catalyze the deamination of phenylalanine to cinnamic acid and could be found in all higher plants^35^,^36^. The PALs from *Bambusa oldhamii* and yeast were selected as candidates because of their different activities when expressed in *E. coli*^28^,^29^. Although 4CLs have been believed to be specific to plants, 4CL from *Streptomyces coelicolor* shows more than 40% identity to plant 4CLs in the amino acid and favored cinnamic acid over *p*-coumaric acid as the substrate^26^. The 4CL from *Petroselinum crispum* displayed the highest pinocembrin production in a previous literature report^9^. Thus, 4CL from *Streptomyces coelicolor* and *Petroselinum crispum* were used for the pathway with PALs. Results showed that the combination of *BO*PAL and *PA*4CL in the pinocembrin biosynthesis pathway was the most suitable configuration to yield high product titers (Table 1). Although the level of cinnamic acid was significantly decreased (*p* < 0.05), the difference in pinocembrin production was not statistically significant (*p* > 0.05). This might be ascribed to the level of gene expression and the burden associated with plasmid propagation^7^. In previous studies, promoters of varying strength and plasmid copy numbers were utilized to adjust the gene expression levels and alleviate the metabolic burden^37^,^38^,^39^. The promoter strength was estimated as T7 = 5, Trc = 1^40^. In addition, the gene copy numbers of pACYCDuet-1(p15A origin), pTrc99A (pBR322 origin), pETDuet-1 (pBR322 origin), and pRSFDuet-1 (RSF origin) that we used were 10, 40, 40 and 100, respectively. After using promoter and plasmid replacement strategy, the strain carrying pTrc-*BO*PAL-*PA*4CL showed a decrease of 85% cinnamic acid than the strain without pathway optimization. Meanwhile, the pinocembrin level displayed a 3.5-fold increase (Fig. 3).

Protein evolution was crucial for the overall product titers, as the site-directed mutagenesis of amino acid 165 (S165M) increased the pinocembrin titer two-fold, whereas the cinnamic acid level remained almost the same (Fig. 4). Directed protein evolution could adapt the enzyme expression to the microbial host system^8^. However, there have been few reports about the CHS from *Glycyrrhiza uralensis*^27^. As part of our experiments performed to better understand the potential explanation underlying the catalysis of CHS, a conserved motif (RLMMYQQGCFAGGTVLR) of CHS from *Arabidopsis thaliana* was revealed by PROSITE. The consensus pattern of CHS was R-[LIVMFYS]-x-[LIVM]-x-[QHG]-x-G-C-[FYNA]-[GAPV]-G-[GAC]-[STAVK]-x-[LIVMF]-[RAL] (Fig. S1), where C is the active site residue. However, S165 of CHS from *Glycyrrhiza uralensis* does not belong to the [FYNA]. F and A were nonpolar amino acid, whereas Y, N and S are all polar uncharged amino acids. The site-directed mutagenesis studies revealed that the positive effect of a nonpolar amino acid (A165 and F165) led to the accumulation of 13.05 and 12.62 mg/L pinocembrin, a 68% and 63% increase, respectively, compared with S165 (Fig. 5). Of note, the mutant strain S165M produced the highest titer of pinocembrin.

To understand the structural consequences of substitutions at the S165, we generated a structural model for CHS using SWISS-MODEL. A structural model for the chalcone synthase of *Alfalfa* (PDB code: 1BI5^41^) served as template for the calculated model because this enzyme shares 94.09% sequence identity with *Glycyrrhiza uralensis* at the protein level. Although the substitution was in close proximity to the assumed active sites of the CHS dimer, its position was not involved in electron transfer reactions. S165 is located at the beginning of a long α-helix and is surrounded by the β-strand at the dimer interface (Fig. S2). The substitution of serine with a hydrophobic amino acid (M, F and A) at this position could achieve a stable structure for CHS. This might ultimately have led to the observed improvement of the CHS activity in *E. coli*. The hydrophobicity values of Pro and Trp were −1.6 and −0.9, which were similar to that of Ser (−0.8)^42^. Their hydrophobicity was lower than that of other nonpolar amino acids. Furthermore, the hydropathicity values of Leu, Ile and Val were 3.8, 4.5 and 4.2, respectively^42^. The substitution with amino acids with high hydrophobicity showed a negative effect on the pinocembrin production. Therefore, the inclusion of hydrophobic amino acids (M 1.9, F 2.8, or A 1.8) was beneficial for CHS activity and pinocembrin production.

The low intracellular malonyl-CoA availability restricts the efficient production of flavonoids in *E. coli*^33^,^43^. Therefore, three strategies for increasing the intracellular malonyl-CoA level were also adopted to achieve a further improvement in pinocembrin production. Overexpression of acetyl-CoA carboxylase^43^,^44^, anaplerosis of the acetate pathway^45^ and inhibition of fatty acid synthesis^34^ have been proven to be efficient means to increase the intracellular malonyl-CoA level. As shown in Table 2, these approaches showed the desired effects as the malonyl-CoA level significantly increased (*p* < 0.05). Due to the increase in malonyl-CoA availability (Table 2), the cinnamic acid production by the engineered strain was decreased compared to the control. Moreover, the pinocembrin production was increased to 40.05 mg/L. Production potential of the optimal strain was tested in a 1.4-L batch fermenter with two-phase pH strategy and a final titer of 67.81 mg/L total pinocembrin was obtained with a productivity of 1.88 mg/L/h. Our present work demonstrated that the accumulation of cinnamic acid could be decreased by a step-wise metabolic engineering strategy.

## Methods

### Strains and plasmids

All constructed strains and plasmids are summarized in Tables S1 and S2. The primers used in this study are listed in Table S3. All constructed plasmids were verified by colony PCR and Sanger sequencing. The detailed pathway construction procedure is described in the Supplementary Information.

### Media and cultivation conditions

*E. coli* seed cultures were grown in Luria-Bertani (LB) medium. The recombinant strains used to produce pinocembrin were cultivated at 37 °C with 200 rpm orbital shaking in MOPS mineral medium. The defined MOPS medium (per liter) contained glucose (5 g), NH_4_Cl (4 g), KH_2_PO_4_ (0.6 g), MOPS (16.74 g), Tricine (0.72 g), FeSO_4_ (3 mg), K_2_SO_4_ (48 mg), CaCl_2_ (0.56 mg), MgCl_2_ (0.11 g) and NaCl (2.93 g). The 1000× trace elements solution (per liter) for MOPS medium contained (NH_4_)_6_(MO_7_)_24_ (37 mg), H_3_BO_3_ (0.25 g), CoCl_2_ (71 mg), CuSO_4_ (25 mg), MnCl_2_ (0.16 g) and ZnSO_4_ (29 mg). Appropriate antibiotics were added at the following concentrations: 100 μg/mL of ampicillin and 50 μg/mL of kanamycin.

A seed inoculum of 500 μL from an overnight 5 mL LB culture was added to a 250 mL flask containing 25 mL MOPS medium for propagation at 37 °C with orbital shaking at 200 rpm. The medium was supplemented with 5 g/L glucose and 4 g/L NH_4_Cl. An additional 25 mL of fresh MOPS medium, 0.60 mM IPTG and 0.50 g/L phenylalanine were added after 6 h of cultivation, and the cultures were incubated at 28 °C with orbital shaking at 200 rpm to induce the protein expression. The cinnamic acid and pinocembrin concentrations were measured after an induction time of 48 h.

### Site-directed mutagenesis

Site-directed mutagenesis was performed using the overlap-extension PCR method with mutant-specific primers (Table S4) containing appropriate base substitution(s). The pUC-CHS plasmid was used as the template. The mutant ORF fragments (CHS mutation) were cloned into the *Nco*I and *Eco*RI sites of the pRSF-CHS-CHI to generate the mutant plasmid.

### Two–phase pH control of batch fermentation for pinocembrin production

Batch fermentation was performed in a 1 L bioreactor using an 800 mL working volume. The strains were cultured in a modified MOPS medium (per liter) containing the following components: glucose (8 g), NH_4_Cl (6 g), KH_2_PO_4_ (0.5 g), MOPS (16.74 g), Tricine (0.72 g), FeSO_4_ (3 mg), K_2_SO_4_ (48 mg), CaCl_2_ (0.56 mg), MgCl_2_ (0.11 g) and NaCl (2.93 g). The 1000× trace elements solution (per liter) for MOPS medium contained (NH_4_)_6_(MO_7_)_24_ (37 mg), H_3_BO_3_ (0.25 g), CoCl_2_ (71 mg), CuSO_4_ (25 mg), MnCl_2_ (0.16 g) and ZnSO_4_ (29 mg). The temperature and agitation were maintained at 37 °C and 250 rpm, respectively. A constant flow of sterile air at 1 vvm was maintained throughout fermentation. After the OD_600_ reached 1.2–1.5, the recombinant cells were induced with 0.8 mM IPTG. Then, 0.83 g/L L-phenylalanine and 2 g/L sodium acetate were added to the medium. In addition, the temperature was maintained at 28 °C. The pH during the first step (from 0–12 h) was kept at 7.0 with 3 M KOH, and the pH during the second step was controlled at 6.5 during hours 12–48.

### Analytical procedures

Cell growth was detected via the optical density measured at 600 nm (OD_600_) using a spectrophotometer (Thermo Scientific, Waltham, MA, USA). To assess the levels of cinnamic acid and pinocembrin, the supernatant was extracted with an equivalent volume of ethyl acetate, vortexed, and centrifuged at 6000 rpm for 3 min at 4 °C. Then, the upper organic layer was removed and evaporated to dryness. The remaining residue was resolubilized with methanol (TEDIA, Fairfield, OH, USA). Samples were quantified by HPLC (Alltech, Deerfield, IL, USA) using an Alltech series 1500 instrument equipped with a prevail C18 reverse-phase column (5 μm, 250 × 4.6 mm; Grace, Deerfield, IL, USA) maintained at 25 °C. For detection, 0.1% acetic acid (solvent A) and acetonitrile supplemented with 0.1% acetic acid (solvent B) were applied as the mobile phases at a flow rate of 1.0 mL min^−1^. The elution was performed according to the following conditions: minute 0–1: 15% B; minutes 1–10: 15% to 40% B; minutes 10–15: 40% to 50% B; minutes 15–25: 50% to 85% B; minutes 25–30: 85% to 15% B; and minute 30–31: 15% B. Products were detected by monitoring the absorbance at 300 nm. Malonyl-CoA was determined by HPLC according to previous procedures^46^.

### Statistical analysis

The experimental data were analyzed for variance by an analysis of variance (ANOVA), followed by Tukey’s post-hoc analysis; mean differences were established by a two-tailed *t*-test at α = 0.05; *p* < 0.05 was considered to indicate a significant difference. The experiments were conducted in triplicate. The values shown represent the means ± standard deviations (SD).

## Additional Information

**How to cite this article**: Cao, W. *et al*. Enhanced pinocembrin production in *Escherichia coli* by regulating cinnamic acid metabolism. *Sci. Rep.* **6**, 32640; doi: 10.1038/srep32640 (2016).

## Supplementary Material

Supplementary Information

## Figures and Tables

**Figure 1:**
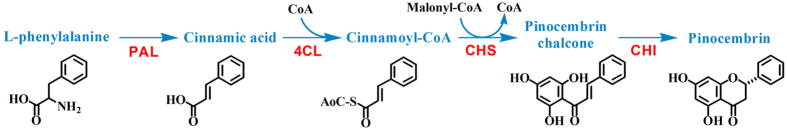
Metabolic pathway for pinocembrin production in *E. coli*. PAL: phenylalanine ammonia lyase, 4CL: 4-coumarate:CoA ligase, CHS: chalcone synthase, CHI: chalcone isomerase.

**Table 1:** Effects of different combinations of different enzyme sources on the cinnamic acid and pinocembrin production by *E. coli* after 48 h in MOPS medium.

Enzyme sources (PAL+4CL)	Cinnamic acid (mg/L)	Pinocembrin (mg/L)
*YE*PAL+*SE*4CL	142.95 ± 5.75	1.71 ± 0.17
*YE*PAL+*PA*4CL	63.62 ± 0.75	1.94 ± 0.25
*BO*PAL+*SE*4CL	20.30 ± 0.85	1.87 ± 0.07
*BO*PAL+*PA*4CL	26.07 ± 4.22	2.34 ± 0.02

^*^Each value is the mean ± standard deviation of three biological replicates.

**Table 2:** Intracellular malonyl-CoA concentrations in different engineered *E. coli* strains.

Strains	Malonyl-CoA (nmol/mg DCW)	Fold change relative to WT
WT	0.41 ± 0.02	1
ACC overexpression	0.89 ± 0.07	2.17
*acs* and ACC overexpression	1.03 ± 0.07	2.51
FabF overexpression	0.69 ± 0.13	1.68
*acs*, ACC, and FabF overexpression	1.36 ± 0.06	3.32

^*^Each value is the mean ± standard deviation of three biological replicates.
