# Supplementary material for: Enhanced pinocembrin production in Escherichia coli by regulating cinnamic acid metabolism
Source: Sci Rep. 2016 Sep 2;6:32640. doi: 10.1038/srep32640 (PMC5009306; doi:10.1038/srep32640)
Supplement: Supplementary Information [file srep32640-s1.pdf]

Enhanced pinocembrin production in *Escherichia coli* by regulating cinnamic acid metabolism

Weijia Cao [1,2], Weichao Ma[1,2,3], Xin wang[1,2], Bowen Zhang[1,2], Xun Cao[1,2], Kequan Chen[1,2*], Yan Li[1,2] & Pingkai Ouyang[1,2]

[1]State Key Laboratory of Materials-Oriented Chemical Engineering, Nanjing 211816, P.R. China

[2]College of Biotechnology and Pharmaceutical Engineering, Nanjing Tech University, Nanjing 211816, P.R. China

[3]College of Bioengineering and Biotechnology, Tianshui Normal University, Tianshui 741001, P.R. China

*Correspondence and requests for materials should be addressed to K.C. (kqchen@njtech.edu.cn)

Construction of plasmids. pET-*YE*PAL was constructed by amplifying *Rhodotorula mucilaginosa* PAL (*YE*PAL) from pET28a-4GS [30] using primers *YE*PAL-F (*Bam*HI) and *YE*PAL-R (*Sac*I) and cloning the resulting product into the *Bam*HI/*Sac*I sites of pETDuet-1. pET-*YE*PAL-*SE*4CL was constructed by amplifying *Streptomyces. coelicolor* A3 (2) 4CL (*SE*4CL) from pET28a-4GS using primers *SE*4CL-F (*Nde*I) and *SE*4CL-R (*Xho*I) and cloning the resulting product into the *Nde*I/*Xho*I sites of pET-*YE*PAL. pET-*BO*PAL was constructed by amplifying *Bambusa oldhamii* PAL (*BO*PAL) from pUC57-*BO*PAL (synthesized by GENEWIZ, Nanjing, China) using primers *BO*PAL-F (*Nco*I) and *BO*PAL-R (*Hind*III) and cloning the resulting product into the *Nco*I/*Hind*III sites of pETDuet-1. pET-*BO*PAL-*SE*4CL was constructed by cloning the *SE*4CL product into the *Nde*I/*Xho*I sites of pET-*BO*PAL. pET-*YE*PAL-*PA*4CL was constructed by amplifying *Petroselinum crispum* 4CL (*PA*4CL) from pUC57-*PA*4CL (synthesized by GENEWIZ, Nanjing, China) using primers *PA*4CL-F (*Nde*I) and *PA*4CL-R (*Xho*I) and cloning the resulting product into the *Nde*I/*Xho*I sites of pET-*YE*PAL. pET-*BO*PAL-*PA*4CL was constructed through the digestion of both pET-*BO*PAL-*SE*4CL and pET-*YE*PAL-*PA*4CL with *Nde*I and *Xho*I, followed by ligation of the appropriate fragments.

pET-CHS was constructed by amplifying *Glycyrrhiza uralensis* CHS from pET28a-4GS [30] using primers CHS-F (*Nco*I) and CHS-R (*Eco*RI) and cloning the resulting product into the *Nco*I/*Eco*RI sites of pETDuet-1. pUC-CHS was constructed through the digestion of both pET-CHS and pUC19 with *Bam*HI and *Eco*RI, followed by ligation of the appropriate fragments. pET-CHS-CHI was constructed by amplifying *Medicago sativa* CHI from pET28a-4GS using primers CHI-F (*Nde*I) and CHI-R (*Xho*I) and cloning the resulting product into the *Nde*I/*Xho*I sites of pET-CHS. pRSF-CHS-CHI was constructed through the digestion of both pET-CHS-CHI and pRSFDuet-1 with *Nco*I and *Xho*I, followed by ligation of the appropriate fragments.

pTrc-*YE*PAL was constructed by amplifying *YE*PAL from pET28a-4GS using primers *YE*PAL-F2 (*Sac*I) and *YE*PAL-R2 (*Xba*I) and cloning the resulting product into the *Sac*I/*Xba*I sites of pTrc99a. pTrc-*SE*4CL was constructed by amplifying *SE*4CL from pET28a-4GS using primers *SE*4CL-F2 (*Bam*HI) and *SE*4CL-R2 (*Hind*III) and cloning the resulting product into the *Bam*HI/*Hind*III sites of pTrc99a. pTrc-*BO*PAL was constructed by amplifying *BO*PAL from pUC57-*BO*PAL by using primers *BO*PAL-F (*Nco*I) and *BO*PAL-R (*Hind*III) and cloning the resulting product into the *Nco*I/*Hind*III sites of pTrc99a. pTrc-*PA*4CL was constructed by amplifying *PA*4CL from pUC57-*PA*4CL by using primers *PA*4CL-F2 (*Eco*RI) and *PA*4CL-R2 (*Pst*I) and cloning the resulting product into the *Eco*RI/*Pst*I sites of pTrc99a.

*YE*PAL-rrnB fragment was amplified from *YE*PAL to rrnB T1 terminator by using *YE*PAL-F2 (*Sac*I) and *YE*PAL-R3 (*Bgl*II). Trc-*SE*4CL-rrnB fragment was amplified from Trc promoter to rrnB T1 terminator by using *SE*4CL-F3 (*Bam*HI) and *SE*PAL-R3 (*Xba*I). Trc-*BO*PAL-rrnB fragment was amplified from *BO*PAL to rrnB T1 terminator using *BO*PAL-F (*Nco*I) and *BO*PAL-R2 (*Bgl*II). Trc-*PA*4CL-rrnB fragment was amplified from Trc promoter to rrnB T1 terminator using *SE*4CL-F3 (*Bam*HI) and *SE*4CL–R3 (*Xba*I). pTrc-*YE*PAL-*SE*4CL was constructed through the digestion of pTrc99a, Trc-*YE*PAL-rrnB and Trc-*SE*4CL-rrnB with *Sac*I/*Xba*I, *Sac*I/*Bam*HI, and *Bgl*II/*Xba*I, followed by ligation of the appropriate fragments. pTrc-*YE*PAL-*PA*4CL, pTrc-*BO*PAL-*SE*4CL and pTrc-*BO*PAL-*PA*4CL were constructed as same as pTrc-*YE*PAL-*SE*4CL.

pACYC-Trc-BOPAL-Trc-PA4CL was constructed by through the digestion of both pTrc-*BO*PAL-*PA*4CL and pACYCDuet-1 with *Nco*I and *Xho*I, followed by ligation of the appropriate fragments. Fused-CHS-CHI was amplified by using Fused-F (*Xba*I) and Fused-R (*Xba*I). pTrc-*BO*PAL-*PA*4CL-CHS-CHI was constructed by in-fusion cloning of pTrc-*BO*PAL-*PA*4CL and

Fused-CHS-CHI.

pRSF-ACC was constructed by amplifying *acc*BC and *dts*R1 from *C. glutamicum* using primers *accBC*-F (*Eco*RV), *accBC*-F (*Kpn*I), *dtsR1*-F (*Kpn*I) and *dtsR1*-R (*Avr*II) and then cloning the resulting product into the *Eco*RV/*Avr*II sites of pRSFDuet-1. pRSF-*acs*-ACC was constructed by amplifying *acs* from *E.coli* using primers *acs*-F (*Bam*HI) and *acs*-F (*Not*I) and cloning the resulting product into the *Bam*HI/*Not*I sites of pRSF-ACC.

pRSF-acs-ACC-CHS(S165M)-CHI was constructed by amplifying CHS(S165M)-CHI from pRSF-CHS(S165M)-CHI using primers Fused-F2 (*Avr*II) and Fused-R2 (*Avr*II) and cloning the resulting product into the *Avr*II sites of pRSF-*acs*-ACC.

Supplementary Table

Table S1 Strains used in this study

Strains	Description	Source or reference
E. coli BL21(DE3)	F^- ompT hsdS$_B$ (r_B^- m_B^-) gal (λ cI857 ind1 sam7 nin5 lacI lacUV5-T7 gene1), dcm(DE3)	Transgen
E. coli Trans-T1	F^- φ80 (lacZ) ΔM15 ΔlacX 74 hsdR(r_k^- m_k^-) ΔrecA 1398endA1 tonA	Transgen
E.coli K-12	Wild-type strain, F^+ rpoS (AM) *rph*-l	CGSC 4401
Corynebacterium glutamicum ATCC 13032	Clone for ACC	ATCC
WT	*E. coli* BL21(DE3) carrying pRSFDuet-1	This study
Ser	*E. coli* BL21(DE3) carrying pTrc-*BO*PAL-*PA*4CL and pRSF-CHS-CHI	This study
ACC overexpression	*E. coli* BL21(DE3) carrying pRSF-ACC	This study
acs and ACC overexpression	*E. coli* BL21(DE3) carrying pRSF-acs-ACC	This study
FabF overexpression	*E. coli* BL21(DE3) carrying pACYC-FabF	[18]
acs, ACC, and FabF overexpression	*E. coli* BL21(DE3) carrying pRSF-acs-ACC and pACYC-FabF	This study
S165M, *acs*↑, ACC↑, FabF↑	*E. coli* BL21(DE3) carrying pTrc-*BO*PAL-*PA*4CL, pRSF-acs-ACC-CHS(S165M)-CHI and pACYC-FabF	This study

Table S2 Plasmids used in this study

Plasmids	Description	Source or reference
pET-28a	T7 promoter, pBR322 ori, , Km^R	Novagen
pETDuet-1	Double T7 promoters, pBR322 ori, Amp^R	Novagen
pACYCDuet-1	Double T7 promoters, P15A ori, Cm^R	Novagen
pRSFDuet-1	Double T7 promoters, RSF ori, Kn^R	Novagen
pUC19	pBR322 ori, Amp^R	Lab collection
pTrc99a	Trc promoter, pBR322 ori, , Amp^R	Lab collection
pET28a-4GS	pET-28a carrying *pal* from *R. mucilaginosa*, *4cl* from *S. coelicolor*, *chs* from *G. uralensis* and *chi* from *M. sativa*	[30]
pET-*YE*PAL-*SE*4CL	pETDuet-1 carrying *pal* from *R. mucilaginosa*, *4cl* from *S. coelicolor*	This study
pET-*YE*PAL-*PA*4CL	pETDuet-1 carrying *pal* from *R. mucilaginosa*, *4cl* from *P. crispum*	This study
pET-*BO*PAL-*SE*4CL	pETDuet-1 carrying *pal* from *B. oldhamii*, *4cl* from *S. coelicolor*	This study
pET-*BO*PAL-*PA*4CL	pETDuet-1 carrying *pal* from *B. oldhamii*, *4cl* from *P. crispum*	This study
pTrc-*YE*PAL	pTrc99a carrying *pal* from *R. mucilaginosa*	This study
pTrc-*SE*4CL	pTrc99a carrying *4cl* from *S. coelicolor*	This study
pTrc-*BO*PAL	pTrc99a carrying *pal* from *B. oldhamii*	This study

pTrc-*PA*4CL	pTrc99a carrying *4cl* from *P. crispum*	This study
pTrc-*YE*PAL-*SE*4CL	pTrc99a carrying *pal* from *R. mucilaginosa*, *4cl* from *S. coelicolor*	This study
pTrc-*YE*PAL-*PA*4CL	pTrc99a carrying *pal* from *R. mucilaginosa*, *4cl* from *P. crispum*	This study
pTrc-*BO*PAL-*SE*4CL	pTrc99a carrying *pal* from *B. oldhamii*, *4cl* from *S. coelicolor*	This study
pTrc-*BO*PAL-*PA*4CL	pTrc99a carrying *pal* from *B. oldhamii*, *4cl* from *P. crispum*	This study
pTrc-*BO*PAL-*PA*4CL-CHS-CHI	pTrc99a carrying *pal* from *B. oldhamii*, *4cl* from *P. crispum*, *chs* from *G. uralensis* and *chi* from *M. sativa*	This study
pACYC-Trc-BOPAL-Trc-PA4CL	pACYCDuet-1 carrying *pal* from *B. oldhamii*, *4cl* from *P. crispum* under Trc promoter	This study
pET-CHS	pETDuet-1 carrying *chs* from *G. uralensis*	This study
pUC-CHS	pUC19 carrying *chs* from *G. uralensis*	This study
pET-CHS-CHI	pETDuet-1 carrying *chs* from *G. uralensis* and *chi* from *M. sativa*	This study
pRSF-CHS-CHI	pRSFDuet-1 carrying *chs* from *G. uralensis* and *chi* from *M. sativa*	This study
pRSF-ACC	pRSFDuet-1 carrying *C. glutamicum accBC* and *dtsR1*	This study

pRSF-*acs*-ACC	pRSFDuet-1 carrying *E. coli* acs and *C. glutamicum accBC* and *dtsR1*	This study
pRSF-acs-ACC-CHS(S165M)-CHI	pRSFDuet-1 carrying *E. coli* acs, *C. glutamicum accBC* and *dtsR1*, *chs* (S165M) from *G. uralensis* and *chi* from *M. sativa*	This study

Table S3 Primers used in this study

Oligonucleotides	Sequences, 5’-3’*
*YE*PAL-F (BamHI)	CGC**GGATCC**GATGGCTCCGTCTGTTG
*YE*PAL-R (SacI)	C**GAGCTC**TTAAGCCATCATTTTAACCAGAACC
*SE*4CL-F (NdeI)	GGAATTC**CATATG**TTCCGTTCTGAATACGCTGAC
*SE*4CL-R (XhoI)	CCG**CTCGAG**TAACGCGGTTCACGCAGCTG
*BO*PAL-F (NcoI)	CATG**CCATGG**GCATGCCACGCGAGGATG
*BO*PAL-R (HindIII)	CCC**AAGCTT**TTAGCAAATCGGCAGCGG
*PA*4CL-F (NdeI)	GGAATTC**CATATG**GGTGACTGCGTTGCTCC
*PA*4CL-R (XhoI)	CCG**CTCGAG**TTATTTCGGCAGGTCACCAG
CHS-F (NcoI)	CATG**CCATGG**TTTCTGTTGCTGAAATCC
CHS-R (EcoRI)	CG**GAATTC**TTAGATAGCAACAGAGTGCAG
CHI-F (NdeI)	GGGAATTC**CATATG**GCTGCTTCTATCACCG
CHI-R (XhoI)	CCG**CTCGAG**TTAGTTACCGATTTTGAAAGCACC
*YE*PAL-F2 (SacI)	C**GAGCTC**ATGGCTCCGTCTGTTGACTC
*YE*PAL-R2 (XbaI)	CTAG**TCTAGA**TTAAGCCATCATTTTAACCAGAACCG
*SE*4CL-F2 (BamHI)	CGC**GGATCC**ATGTTCCGTTCTGAATACGC
*SE*4CL-R2 (HindIII)	CCC**AAGCTT**TTAACGCGGTTCACGCAG
*PA*4CL-F2 (EcoRI)	CCG**GAATTC**ATGGGTGACTGCGTTGCTCC
*PA*4CL-R2 (PstI)	AAAA**CTGCAG**TTATTTCGGCAGGTCACCAGAAGC
*YE*PAL-R3 (BglII)	GGA**AGATCT**ATTTGTCCTACTCAGGAGAGCGTTC
*SE*4CL-F3 (BamHI)	CGC**GGATCC**TTGACAATTAATCATCCGGCTCG

*SE*PAL-R3 (XbaI)	CTAG**TCTAGA**ATTTGTCCTACTCAGGAGAGCGTTC
*BO*PAL-R2 (BglII)	GGA**AGATCT**ATTTGTCCTACTCAGGAGAGCGTTC
Fused-F (XbaI)	CTGAGTAGGACAAAT**TCTAGA**GGATCTCGACGCTCTCCCTTATGC
Fused-R (XbaI)	TGCCTGCAGGTCGAC**TCTAGA**GACCGTGTGCTTCTCAAATGCCTGAG
accBC-F (EcoRV)	GGCC**GATATC**GGTGTCAGTCGAGACTAGGAAGATCAC
accBC-F (KpnI)	CGG**GGTACC**CTTGATCTCGAGGAGAACAACG
dtsR1-F (KpnI)	CGG**GGTACC**ATGACCATTTCCTCACCTTT
dtsR1-R (AvrII)	CGG**CCTAGG**TTACAGTGGCATGTTGCC
acs-F (BamHI)	CG**GGATCC**GATGAGCCAAATTCACAAACACAC
acs-F (NotI)	AAGGAAAAAA**GCGGCCGC**TTACGATGGCATCGCGATAG
Fused-F2 (*Avr*II)	CGGCAACATGCCACTGTAA**CCTAGG**CGACTCCTGCATTAGG
Fused-R2 (*Avr*II)	CAGCGGTGGCAGCAG**CCTAGG**CAGCAGCGGTTTCTTTACCAG

*Bold and underlined letters are restriction enzyme cut sites.

Table S4 mutant-specific primers in this study

Oligonucleotides	Sequences, 5’-3’
Phe-F	GAACGGTACCACCAGCGAAGCAACCCTGCTGGTAC
Phe-R	TCGCTGGTGGTACCGTTCTGCGTCTGGCTAAAGAC
Asp-F	GAACGGTACCACCAGCATCGCAACCCTGCTGGTAC
Asp-R	GATGCTGGTGGTACCGTTCTGCGTCTGGCTAAAG
Lys-F	GAACGGTACCACCAGCTTTGCAACCCTGCTGGTAC
Lys-R	AAAGCTGGTGGTACCGTTCTGCGTCTGGCTAAAG
Ala-F	GAACGGTACCACCAGCTGCGCAACCCTGCTGGTAC
Ala-R	GCAGCTGGTGGTACCGTTCTGCGTCTGGCTAAAGAC
Val-F	GAACGGTACCACCAGCAACGCAACCCTGCTGGTAC
Val-R	GTTGCTGGTGGTACCGTTCTGCGTCTGGCTAAAGAC
Leu-F	GAACGGTACCACCAGCCAGGCAACCCTGCTGGTAC
Leu-R	CTGGCTGGTGGTACCGTTCTGCGTCTGGCTAAAGAC
Pro-F	GAACGGTACCACCAGCCGGGCAACCCTGCTGGTAC
Pro-R	CCGGCTGGTGGTACCGTTCTGCGTCTGGCTAAAGAC
Trp-F	GAACGGTACCACCAGCCCAGCAACCCTGCTGGTAC
Trp-R	TGGGCTGGTGGTACCGTTCTGCGTCTGGCTAAAGAC
Met-F	GAACGGTACCACCAGCCATGCAACCCTGCTGGTAC
Met-R	ATGGCTGGTGGTACCGTTCTGCGTCTGGCTAAAGAC
Ile-F	GAACGGTACCACCAGCAATGCAACCCTGCTGGTAC
Ile-R	ATTGCTGGTGGTACCGTTCTGCGTCTGGCTAAAGAC

Supplementary Figure

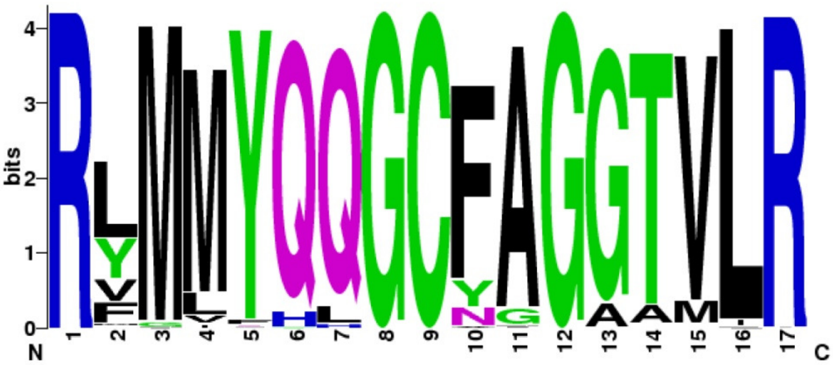

Fig. S1 The sequence logo of chalcone and stilbene synthases by PROSITE. 9th C: the active site residue.

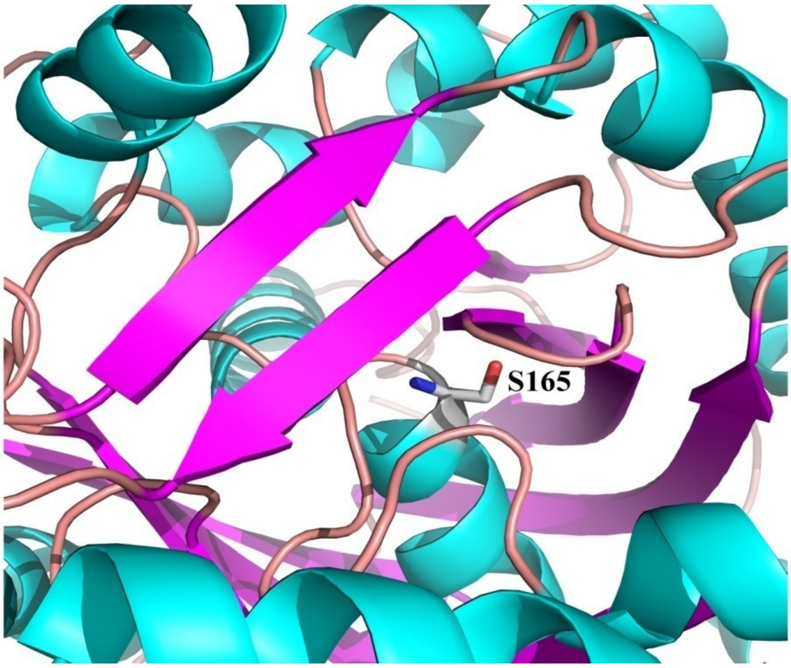

Fig. S2 A structure mode of *G. uralensis* CHS by SWISS-MODEL. S165: the mutation site
